# The Potency of Hyaluronan of Different Molecular Weights in the Stimulation of Blood Phagocytes

**DOI:** 10.1155/2010/380948

**Published:** 2011-02-09

**Authors:** Barbora Safrankova, Silvie Gajdova, Lukas Kubala

**Affiliations:** ^1^Institute of Biophysics, The Academy of Sciences of the Czech Republic, v. v. i., Královopolská 135, 612 65 Brno, Czech Republic; ^2^Faculty of Science, Masaryk University, Kotlářská 2, 611 37 Brno, Czech Republic

## Abstract

The regulatory functions of glycosaminoglycan hyaluronan (HA) are suggested to be dependent on its molecular weight (MW). Proinflammatory and stimulatory effects are proposed mainly for the low MW HA. However, the complex response of blood phagocytes to HA of different MW is unclear. Herein, the effects of highly purified HA of precisely defined MW (52, 250, and 970 kDa) on human blood phagocytes were tested. All MW HA activated blood phagocytes, including the spontaneous production of ROS, degranulation, and the production of tumor necrosis factor alpha, with low MW HA 52 kDa having the highest potency and high MW HA 970 kDa having the lowest potency. Interestingly, HA inhibited ROS production stimulated by opsonized zymosan particles and, in contrast, potentiated starch-activated ROS production, mostly independent of MW. Data showed a significant effect of HA of different MW on blood phagocytes, including high MW HA.

## 1. Introduction

Inflammation is a complex process underlying the pathogenesis of numerous chronic and acute diseases. Intensive tissue remodeling occurs at the site of inflammation. The extracellular matrix is degraded and replaced by a newly synthesized one that contributes to the tissue healing process. One of the main components of the extracellular matrix is hyaluronan (HA). HA in its native form is a high-molecular-weight (HMW) linear glycosaminoglycan reaching a size of 6 to 8 MDa [1–3]. HA binding at the cell surface is a complex interaction of multivalent binding events affected mainly by the quantity, density, and activation state of cell surface HA receptors, including CD44 and Toll-like receptors 2 and 4 [2, 4–6]. HA undergoes intensive metabolism in the organism dependent on tissue [7]. In particular, inflammation significantly increases HA degradation to low-molecular-weight (LMW) fragments. Despite their simple primary structure, HA molecules have been reported to have extraordinarily wide-ranging and often opposing biological functions [2, 3]. HMW HA is widely reported to possess antiangiogenic, anti-inflammatory, and immunosuppressive properties. On the other hand, smaller fragments of HA polymers (LMW HA) are believed to be proinflammatory and angiogenic [1, 6]. 

Among the most important cells in the inflammatory process are phagocytes that include tissue-specific macrophages and polymorphonuclear leukocytes (PMNL), particularly neutrophil granulocytes [8]. Phagocytes play a critical role in controlling acute and chronic tissue inflammation, through the release of a variety of mediators, including cytokines, chemokines, growth factors, and reactive oxygen species (ROS) [8–10]. ROS production by phagocytes is connected with the so-called “oxidative burst,” which is a key process against invading pathogens [8, 10]. The oxidative burst is closely related with degranulation of blood phagocytes accompanied by an increase in surface receptor expression [11]. Thus, the ROS production by blood phagocytes and the surface expression of receptors allow us to determine phagocyte activation induced by various stimuli and to predict physiological consequences of the activation [11–13].

In general, there is a suggested stimulatory effect of LMW HA and an inhibitory effect of HMW HA on blood phagocytes, similar to other cell types [14]. In different studies, various authors evaluated the effect of HA on blood phagocyte functions, particularly phagocytosis [15–20]. However, a detailed description of blood phagocyte response to HA of different MW in complex environment of whole blood is absent. Furthermore, the current data are often contradictory, dependent on the employed model system and the selected MW HA. The main question raised by many of these previous studies is the origin and purity of the HA preparations used. 

In the present study, the potential of HMW HA (970 kDa), middle-sized MW (250 kDa), and LMW HA (52 kDa) to activate blood phagocytes was tested *in vitro*. In contrast to other studies, we used highly purified HA of a pharmacological grade with a low polydispersity index. Phagocyte activity was evaluated based on the determination of the ROS production, the degranulation, and the production of tumor necrosis factor alpha. Effects of selected MW of HA were also tested in the presence of collagen, as an abundant component of the extracellular matrix in tissue. Interestingly, all MW HA increased the overall activation of blood phagocytes. In addition, HA inhibited ROS production stimulated by opsonized zymosan particles (OZP), which was in contrast to the potentiation of starch-activated ROS production by HA. In general, our data provide information about the role of HA in the activation of blood phagocytes.

## 2. Materials and Methods

### 2.1. HA Preparation

**Figure 1:**
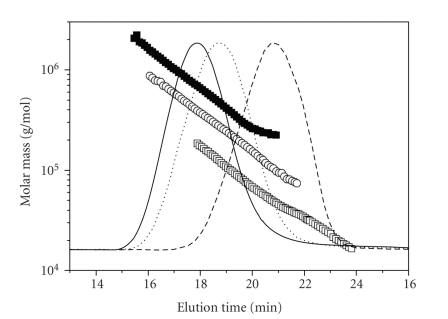
Light scattering chromatograms (lines) and molar mass versus elution time plot (symbols) from SEC-MALS analysis for 52 kDa HA preparation (dashed line, □), 250 kDa HA preparation (dotted line, ○), and 970 kDa preparation (solid line, ■).

HA of Streptococcus equi biotechnological origin and pharmaceutical grade (Contipro C, Dolni Dobrouc, Czech Republic) was used [21]. To prepare HA of a particular molecular weight (MW), 1% water solution of 0.97–2.33 MDa HA was degraded by acid hydrolysis, utilizing HCl at pH 3.8, 100°C, for different time periods. Each product was isolated by ethanol precipitation, followed by repeated washing with isopropanol and centrifugation. The precipitate was dissolved in water (1% solution), and the spray dried. SEC-MALS analysis was performed with the Agilent 1100 Series chromatography system (Santa Clara, CA, USA) equipped with the following column system: PL aquagel-OH Mix and PL aquagel-OH 30 (300 × 7.5 mm, 8 *μ*m; Polymer Laboratories). The eluent (0.1 M sodium phosphate buffer pH 7.5) was monitored using a DAWN-EOS multiangle laser light scattering photometer (18-angle, Wyatt Technologies Corporation) and refractive index detector Optilab rEX (Wyatt Technologies Corporation). Data acquisition and MW calculations were performed using ASTRA V software, Version 5.3.2.15. The flow rate of the mobile phase was maintained at 0.8 mL/min. The specific refractive index increment (dn/dc) of 0.155 mg/mL was used for sodium HA. SEC-MALS analysis showed both MW and MW distribution [22]. The normalized light scattering chromatograms with depicted MW (molecular mass in the figure legend) distributions are shown in Figure 1. None of the samples contained HA oligomers (HA chains with molar mass below 10 kDa). The MW distributions were very narrow: LMW HA with 52 000 g/mol (52 kDa) showed MW/Mn 1.14, middle-size HA with 250 000 g/mol (250 kDa) showed MW/Mn 1.35, and HMW HA with 970 000 g/mol (970 kDa) showed MW/Mn 1.34. The presence of endotoxins was tested using the PyroGene Recombinant Factor C Endotoxin Detection System (Cambrex, USA), which did not detect any significant amount of endotoxin (less than 0.01 EU/mL). Stock solutions of HA preparations (1 mg/mL) were prepared fresh in HBSS.

### 2.2. Materials

Unless otherwise stated all other reagents were purchased from Sigma-Aldrich and were p.a. grade and higher (USA). The stock solution of 10 mM luminol (5-amino-2,3-dihydro-1,4-phthalazinedione) (Invitrogen, USA) was prepared in 0.2 M sodium borate buffer, pH 9.0 (1.24 g of H_3_BO_3_ and 7,63 g of Na_2 _B_4 _O_7_ · 10H_2_O in 1 liter of redistilled water). Phorbol 12-myristate 13-acetate (PMA) was dissolved in dimethyl sulfoxide to obtain 3.25 *μ*mol/mL stock solution. OZP were prepared by opsonisation of zymosan A from *Saccharomyces cerevisie* by human serum to obtain 5 mg/mL stock solution, as described previously [10]. Rice starch grains (10 mg /mL, Amylum oryzae, a local pharmacy) were prepared in Hanks balanced salt solution (HBSS), pH 7.4.

### 2.3. Blood Samples Preparation and Experimental Layout of the Incubation of Samples with HA

Blood samples were obtained from healthy volunteers who had given informed consent. Whole blood was diluted 1 : 2 (vol/vol) with RPMI (PAN, Germany). The stock solutions of different MW HA (52; 250 and 970 kDa) were added to the diluted blood to obtain final concentrations of 100 *μ*g/mL and 10 *μ*g/mL. The concentrations were selected in order to be on the lower limit of the range of concentrations used by other authors [15–20, 23–27]. The lower concentration of HA 10 *μ*g/mL was, in our preliminary experiment, shown to be on the border of having any significant effect on blood phagocytes. A concentration of 1 *μ*g/mL, which is higher than the physiological concentration in human blood [7, 28], did not possess any effect on blood phagocyte ROS production or degranulation according to our preliminary experiments on a limited number of samples (data not shown). Further, selected samples were mixed with collagen (final concentration 0.16 mg/mL; Ultrapure collagen solution from bovine skin, Sigma-Aldrich), together with HA. HBSS was used instead of HA or collagen as a control, to adjust the same reaction volume for all samples. To determine the effects of HA on the oxidative burst of blood phagocytes, the ROS production was measured immediately or after 1.5 h incubation at 37°C, prior to determining their oxidative burst. To determine the effects of HA on PMNL degranulation, the surface expression of the receptors specific for particular granules was determined 30 minutes and 90 minutes after addition of HA. To determine the effects of HA on TNF-*α* production, the samples were incubated for 2.5 and 6 h with HA. The time points were selected based on our previous studies [9, 29].

### 2.4. ROS Production (Oxidative Burst) Determination by Chemiluminescence (CL)

Luminol amplified chemiluminescence (CL) was measured using a microplate luminometer Orion II (Berthold Detection System GmbH, Germany). The principle of the method is based on luminol interaction with phagocyte-derived ROS, which results in large, measurable amounts of light [10]. Briefly, the reaction mixture consisted of 100 *μ*l of diluted blood, the tested substances (HA, collagen, or an appropriate amount of buffer), 1 mM luminol, and one of the activators (final concentrations PMA −97.6 *μ*g/mL, OZP −0.4 mg/mL, or starch 1 mg/mL). The assays were run in duplicates. Spontaneous CL measurements in samples containing 100 *μ*l of diluted blood and all other substances, but none of the activators, were included in each assay. The CL emission was followed for 60 min at 37°C. The integral value of the CL reaction, which represents the total ROS production by blood phagocytes, was evaluated, and final data were recalculated as a percentage of the untreated control (100%).

### 2.5. Determination of PMNL Degranulation Based on Surface Expression of Granule-Specific Receptors

Following receptors which surface expression increases significantly upon phagocyte degranulation were selected: complement receptors 1 (CD35), mostly stored in secretory vesicles, complement receptors 3 (CD11b), stored both in specific and gelatinase granules, and CD66, stored primarily in specific granules [11, 13, 30]. Diluted blood was incubated with HA of different MW in concentrations of 100 *μ*g/mL and 10 *μ*g/mL, and in the absence and presence of collagen (as described above), at 37°C for 30 and 120 minutes. Next, samples were incubated with mouse anti-CD66b-FITC (BD Biosciences, USA), mouse anti-CD35-FITC (clone E11, Ancell Corporation, USA), mouse anti-CD11b-FITC (clone ICRF44) (Ancell Corporation), or appropriate isotype controls (BD Biosciences and Ancell Corporation, USA) for 15 minutes at room temperature (RT), then fixed by 4% formaldehyde in PBS for 15 minutes at RT, as described previously [29]. Red blood cells were lysed using distilled water for the subsequent 10 minutes at RT. After centrifugation (300 g, 7 minutes), the remaining cells were resuspended in cold PBS and kept on ice until the assessment of fluorescence by flow cytometer FACSCalibur (BD Biosciences). Ten thousand granulocytes, selected on the basis of their typical scattering characteristics, were analyzed. The geometric mean of the relative fluorescence unit was quantified, and final data were recalculated as a percentage of the untreated control (100%).

### 2.6. Detection of Proinflammatory Cytokine TNF-*α* by ELISA

To determine TNF-*α* production, diluted blood was incubated with HA of a different MW in concentrations of 100 *μ*g/mL and 10 *μ*g/mL, and in the absence and presence of collagen (as described above), at 37°C for 2.5 h and 6 h. After centrifugation (300 g, 10 minutes, RT), supernatant was obtained and TNF-*α* concentrations in the supernatant were detected by ELISA, according to the manufacturer's instructions for TNF-*α* DuoSet ELISA Development System (R&D Systems, Inc., USA). The absorbance was measured at 450 nm against a reference wavelength of 620 nm, using a microtiter plate reader (SLT Rainbow spectrophotometer, Tecan, Carlsheim, Germany). The results were recalculated to pg/mL, and final data were expressed as a percentage of untreated control (100%).

### 2.7. Statistical Analysis of Data

At least four independent repetitions were performed for each experimental setup. All data are reported as a percentage of the control without HA, and all particular values are displayed by means of different symbols together with the median value displayed as a bold line. Nonparametric Friedman ANOVA (StatSoft, USA) was applied to compare differences among the control and treated groups. A *P*  value ≤.05 was considered significant: (∗) statistical significance to control, (#) statistical significance to sample incubated with 52 kDa HA, and ($) statistical significance between samples incubated with 250 kDa and 970 kDa HA.

## 3. Results

### 3.1. Effects of Different MW HA on Blood Phagocyte ROS Production

**Figure 2:**
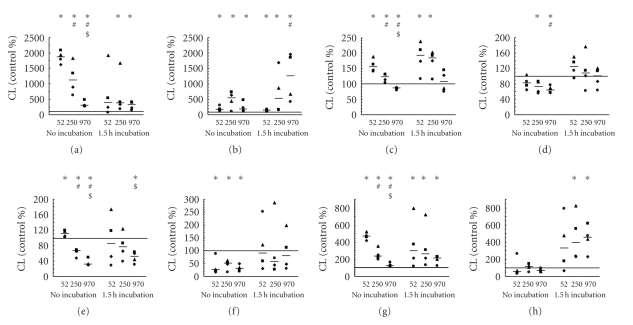
Effect of different MW HA (100 *μ*g/mL) on spontaneous (a, b), PMA-activated (c, d), OZP-activated (e, f), and starch-activated (g, h) ROS production measured in diluted whole blood by luminol-enhanced chemiluminescence in absence (a, c, e, g) and presence of collagen (0.16 mg/mL) (b, d, f, h), immediately or after 1.5 h pre-incubation after addition of HA. The bold line represents the median, and data points from parallel measurements are presented by the same symbol. Statistically significant differences among different groups (*P* < .05) are marked by different symbols: (∗) statistical significance to control, (#) statistical significance to sample incubated with 52 kDa HA, and ($) statistical significance between samples incubated with 250 kDa and 970 kDa HA.

 The effects of different MW HA (100 *μ*g/mL) on blood phagocytes were tested in samples containing diluted whole blood and diluted whole blood with the addition of collagen. All MWs of HA significantly stimulated the spontaneous production of ROS by human whole blood, with LMW HA having a significantly higher effect, middle-sized MW HA having a significantly smaller effect, and HMW HA having the lowest stimulatory effect (Figure 2(a)). The stimulatory potential of all MW HA was exhausted after 1.5 h incubation, with significantly increased ROS production only in samples with middle and high MW HA. In the presence of collagen, all MW HA stimulated spontaneous ROS production without a significant difference among MW HA measured without preincubation (Figure 2(b)). Interestingly, spontaneous ROS production was still significantly higher in samples containing different MW HA, when compared to the control, after pre-incubation with the most potent effect of HMW HA.

Phagocytes can be stimulated in the presence of HA by various activators to cause the HA potential to costimulate or to inhibit blood phagocyte reaction to these activators. Among these activators are OZP, which activate an oxidative burst of phagocytes by binding to the opsonine receptors. This induces a signal transduction that leads to the activation of NADPH oxidase, the key enzyme of the oxidative burst. Starch-mediated activation is dependent on surface receptors recognizing glucan and other types of polysaccharide structures. In contrast, soluble stimuli such as phorbols directly lead to the stimulation of signaling molecules, including protein kinase C involved in these signal transduction pathways [10]. All these selected activators PMA, OZP, and starch grains induced a significant increase in CL (data not shown). Both LMW HA and middle-sized MW HA increased PMA-activated ROS production, in contrast to HMW HA, which significantly decreased PMA-activated ROS production (Figure 2(c)). Similarly, LMW HA and middle-sized HA significantly potentiated PMA-activated ROS production, compared to the control and HMW HA samples after 1.5 h pre-incubation (Figure 2(c)). Interestingly, in the presence of collagen, both middle-sized HA and HMW HA decreased PMA-activated ROS production, which was significant in samples measured without pre-incubation (Figure 2(d)). LWM HA did not affect PMA-activated ROS production either in samples measured immediately and after 1.5 h pre-incubation. 

Subsequently, the modulation of OZP-activated ROS production by HA of different MW was evaluated. OZP-activated ROS production was slightly, however, significantly, potentiated by LMW HA, in contrast to the reduction of OZP-activated ROS production by middle-sized and HMW HA (Figure 2(e)). The reduction effect of HMW HA was also significant after 1.5 h pre-incubation. In the presence of collagen, all MW HA significantly decreased OZP-activated ROS production in samples measured without pre-incubation (Figure 2(f)). No MW HA significantly modified OZP-activated ROS production, when compared to the control after 1.5 h pre-incubation. 

Finally, the effects of HA of different MW on starch-activated ROS production were determined. All tested samples of HA revealed a stimulatory effect on starch-activated ROS production, determined both with and without 1.5 h pre-incubation (Figure 2(g)). Significantly, LMW HA was revealed to have the highest potency to stimulate starch-activated ROS production, followed by middle-sized MW HA, and finally by HMW HA, which had the lowest potency in samples without pre-incubation. In contrast, a stimulatory effect was observed in the presence of collagen only with samples measured after pre-incubation, and it was significant only for middle-sized and HMW HA (Figure 2(h)). 

**Figure 3:**
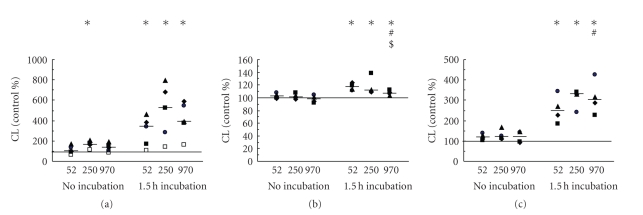
Effect of different MW HA (10 *μ*g/mL) on spontaneous (a), OZP-activated (b), and-starch activated (c) ROS production measured in diluted whole blood by luminol-enhanced chemiluminescence, immediately or after 1.5 h pre-incubation after addition of HA. The bold line represents the median, and data points from parallel measurements are presented by the same symbol. Statistically significant differences among different groups (*P* < .05) are marked by different symbols: (∗) statistical significance to control, (#) statistical significance to sample incubated with 52 kDa HA, and ($) statistical significance between samples incubated with 250 kDa and 970 kDa HA.

To further evaluate the effects of different MW HA on human blood phagocytes ROS production, particularly the controversial effects on OZP and starch-activated ROS production, subsequent tests were performed with a lower concentration of HA 10 *μ*g/mL. Only middle-sized HA in this lower concentration induced spontaneous ROS production (Figure 3(a)). In contrast, all tested MW HA induced spontaneous ROS production in samples pre-incubated for 1.5 h. Interestingly, in contrast to data obtained with higher HA concentrations, OZP-activated ROS production was not significantly modulated in samples measured without pre-incubation and was even potentiated in samples measured after 1.5 h pre-incubation (Figure 3(b)). Similarly, starch-activated ROS production was increased by all tested MW HA, with the highest potency of HMW HA in samples measured after 1.5 h pre-incubation (Figure 3(c)).

### 3.2. Expression of Surface Receptors—Degranulation

The effect of HA (100 *μ*g/mL) on the degranulation of phagocytes was determined on the basis of the changes of surface expression of receptors that are primarily stored in phagocytes granules. 

**Figure 4:**
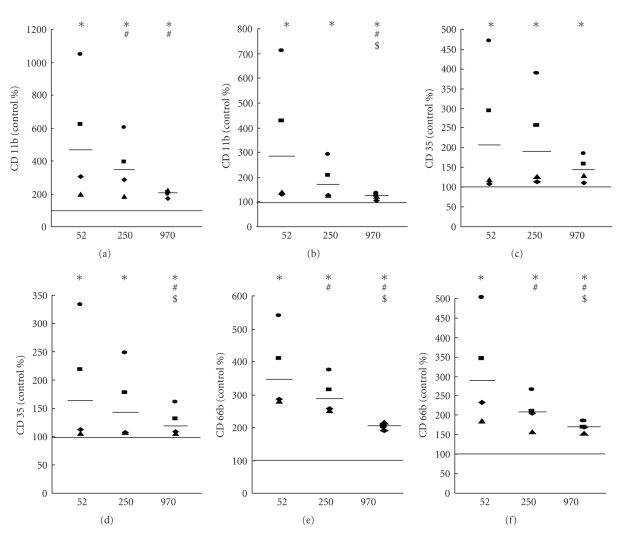
Effect of different MW HA (100 *μ*g/mL) on the surface expression of CD11b (a, b), CD35 (c, d), and CD66b (e, f), determined after incubation for 30 minutes or 120 minutes after addition of HA in absence (a, c, e) and presence of collagen (0.16 mg/mL) (b, d, f). The bold line represents the median, and data points from parallel measurements are presented by the same symbol. Statistically significant differences among different groups (*P* < .05) are marked by different symbols: (∗) statistical significance to control, (#) statistical significance to sample incubated with 52 kDa HA, and ($) statistical significance between samples incubated with 250 kDa and 970 kDa HA.

All MW HA induced an increase in CD11b expression after 120 minutes incubation periods, independent of the presence of collagen (Figures 4(a) and 4(b)). Interestingly, this effect was strongly dependent on MW HA, and significantly highest for LMW HA, lower for middle-sized MW HA, and lowest for HMW HA, in both the absence and presence of collagen (Figures 4(a) and 4(b)). The increase in CD11b surface expression was already observed after 30 min of incubation in both the absence and presence of collagen—however, without any significant differences among MW of HA (data not shown). 

All MW HA induced an increase in CD35 expression after incubation periods of both 30 minutes (data not shown) and 120 minutes (Figure 4(c)), which was not significantly dependent on MW HA. The effect on CD35 expression was similar in the presence of collagen; the most potent effect was observed with LMW HA in samples incubated for 120 minutes (Figure 4(d)).

Similarly, all MW HA induced an increase in CD66b expression after incubation periods of both 30 minutes (data not shown) and 120 minutes (Figures 4(e) and 4(f)). This effect was again strongly dependent on MW HA and significantly highest for LMW HA, lower for middle-sized MW HA, and lowest for HMW HA, in both the absence and presence of collagen (Figures 4(e) and 4(f)). 

We also evaluated the effect of the lower HA concentration of 10 *μ*g/mL. Interestingly, none of the tested MW HA induced any significant increased expression of CD11b and CD35, either after 30 minutes or 120 minutes incubation (data not shown). The only significant effects were observed while evaluating CD66b surface expression after 120 minutes of incubation. In this case a slight, however, significant, expression of CD66b was induced by 250 kDa HA (median 145%, max 165%, min 108%) and 970 kDa HA (median 129%, max 151%, min 109%).

### 3.3. Production of Inflammatory Cytokine TNF-*α*

**Figure 5:**
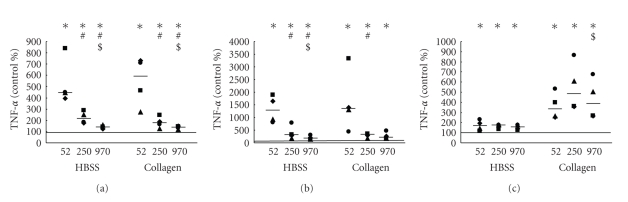
Effect of different MW HA (100 *μ*g/mL—a, b and 10 *μ*g/mL—c) on TNF-*α* production determined in absence and presence of collagen (0.16 mg/mL) 2.5 h (a) and 6 h (b, c) after addition of HA. The bold line represents the median, and data points from parallel measurements are presented by the same symbol. Statistically significant differences among different groups (*P* < .05) are marked by different symbols: (∗) statistical significance to control, (#) statistical significance to sample incubated with 52 kDa HA, and ($) statistical significance between samples incubated with 250 kDa and 970 kDa HA.

TNF-*α* production by whole blood in response to different MW HA was determined as a marker of the overall activation of blood leukocytes to produce inflammatory mediators. All MW HA (100 *μ*g/mL) induced production of TNF-*α*, both after 2.5 h and 6 h (Figures 5(a) and 5(b)). Interestingly, this effect was strongly dependent on MW HA, and significantly highest for LMW HA, lower for middle-sized MW HA, and lowest for HMW HA, in both the absence and presence of collagen (Figures 5(a) and 5(b)). The effect of different MW was less significant in the presence of collagen after 6 h incubation (Figure 5(b)). Interestingly, a significant increase in TNF-*α* production was also observed with a lower concentration 10 *μ*g/mL after 6 h incubation (Figure 5(c)).

## 4. Discussion

HA preparations of all tested MW induced the complex activation of blood phagocytes, with LMW having the highest potency, middle-sized MW HA having the next highest potency, and HMW HA having the lowest potency. The modulation of phagocyte activation by OZP, starch, and PMA was not generally dependent on MW of HA; however, it was modified by the presence of collagen. 

Interestingly, all MW HA stimulated spontaneous production of ROS production, with the most significant effect observed with LMW HA. The increased ROS production was accompanied by the increase in CD11b, CD35, and CD66 surface expression, a marker of degranulation, and production of TNF-*α*. Correspondingly to our study, a significant increase in CD11b expression on PMNL induced by both LMW HA and HMW HA (100 *μ*g/mL) after incubation in whole blood has been observed by Krasiński et al. [27]. Similarly to our observation of the TNF-*α* induction, IL-6 and MCP1 were produced by isolated peripheral blood mononuclear cells in response to LWM HA but not HMW HA [14]. Moreover, these authors showed involvement of HA receptor CD44 in this process employing anti-CD44 inhibition antibody that inhibited the response of cell to LMW HA. In our study, we also evaluated the role of CD44 in the described effects by employing anti-CD44 antibody with a suggested inhibitory effect (purified anti-mouse/human CD44 Antibody, clone IM7, Biolegend, USA). Contrary to our expectations, the application of this antibody induced significant dose-dependent activation of blood phagocytes (data not shown). An appropriate isotype control did not show any significant effect. This discrepancy could account for direct activation of CD44 receptor by anti-CD44 antibody in a complex system as is whole blood.

Next, we focused on the modulation of blood phagocyte response to other activators by HA of different MW. HA of all MW mostly inhibit the response of blood phagocytes to soluble activator PMA. It could be speculated that HA of different MW can inhibit the binding of soluble stimuli to the phagocyte surface and thus can reduce PMA-induced ROS production. In a similar model, Krasiński et al. also found a significant reduction of phagocyte ROS production in response to soluble activators f-Met-Leu-Phe and PMA by HMW HA (100–500 *μ*g/mL) [27]. Furthermore, various authors in studies employing the isolated phagocytes found suppression of phagocyte responses to PMA, liposacharide, and f-Met-Leu-Phe by HA of various MW in a wide range of concentrations [17, 24–26]. In the case of the OZP-activated ROS production, which is dependent primarily on the binding of OZP to cell surface opsonine receptors, all MWs of HA mostly inhibited the activation. This suggests the inhibition of the interaction among OZP and phagocytes receptors recognizing OZP by HA independent of HA MW. In contrast, in the case of the starch-activated ROS production, which is primarily dependent on the binding of starch grains to surface receptors recognizing glucan and other types of polysaccharide structures, HA preparations showed activation potential. Previously, employing heparinized human whole blood as a model system, both HMW HA (2 MDa) and middle-size MW HA (0.2 MDa) stimulated particle stimulated phagocyte ROS production [23]. Various other studies employing isolated blood phagocytes in a less complex environment than whole blood brought inconclusive results. They showed HA-mediated potentiation [15, 19, 20, 23, 31], HA-mediated inhibition [15–17], or even a lack of HA effect [16, 18] on the activation of blood phagocytes by particular stimuli or the phagocytosis of both unopsonised and opsonized particles or bacteria. 

Controversial reports about HA effects on phagocytes could account for several discrepancies. One of the reasons could be the employment of a different range of HA concentrations. Some of these studies showed that the higher concentrations of HA possess inhibitory effects, in contrast to lower concentrations which possess stimulatory effects [15, 16, 23]. However, comparing the above-cited studies, the inhibitory concentrations, the concentrations without effect, and the concentrations with stimulatory effects are overlapping. In our study, we employed HA concentrations that were significantly higher compared to the physiological HA concentrations in blood (10 and 100 *μ*g/mL) [7, 28]. However, the employed concentrations could be theoretically reached locally after the adhesion of phagocytes onto the glycocalyx of endothelial cells and after diapedesis of phagocytes in the tissues. Of interest is also the comparison of employed concentrations of different HA preparations expressed as moles per volume when 52 kDa HA was 1.92 × 10^−6^ mol/l, 250 kDa Ha was 0.40 × 10^−6^ mol/l, and 970 kDa was 0.10 × 10^−6^ mol/l for the higher tested concentration (100 *μ*g/mL). Thus, from this perspective, the concentration of LMW HA tested in this study was more than one-order higher than that used for HMW HA. 

Another reason for the inconsistent results observed by various authors could be differences in the purity of HA MW, including the presence of shortened HA polymers. We employed HA with precisely defined MW and polydispersity. The definition of low, middle, and high MW HA is broad and various authors employed HA of different MW as LMW, middle-size, and HMW HA. Compared to our study, particularly MW of HMW HA could be significantly higher reaching 3-4 MDa. Since our data showed the significance of MW in the direct activation of blood phagocytes by HA, we can suggest that these differences among various authors can also contribute to the inconsistent results obtained by these authors. However, we did not observe any significant effect of MW on the modulation of phagocyte response to OZP or starch. Similarly to our study, the same effects on blood phagocyte activation by particular stimuli have been reported for a wide range of HA MW from HMW to middle-size MW by Hakansson and Venge [31]. In contrast, Tamoto et al. found that HA (0.5–5 mg/mL) inhibited OZP and polystyrene latex particle phagocytosis when investigating guinea pig phagocytes in a dose- and MW-dependent manner [17]. 

The effect of HA on blood phagocytes was tested both in diluted whole blood and in diluted whole blood with the addition of collagen. The presence of collagen should partly mimic the pathological conditions within tissues where HA is in a complex with other components of an extracellular matrix. The presence of collagen did not significantly modulate the effect of different MW HA except that HA potentiated starch-activated ROS production particularly when samples were coincubated with collagen. This suggests that the presence of HA increased the ability of blood phagocytes to interact with starch, and collagen potentiates these effects. This phenomenon is supported by the observations of Hakansson and Venge who suggested that fibronectin, another principal molecule of the extracellular matrix, is key for HA stimulatory effects on phagocytes, particularly HA-mediated stimulation of PMN migration [31, 32].

Interestingly, our findings presented herein are in direct contrast with our previous findings when testing the effects of the same HA preparations on various mouse macrophage cell lines [21]. In that case, we did not observe any significant activation of mouse macrophages, either alone or in combination with costimulators. This suggests that different mechanisms are involved in the activation of blood phagocytes in the complex environment of whole blood. Previously tested macrophage cell lines are highly sensitive to stimulation by any bacterial contaminations or inflammatory mediators, which suggests the specificity of the effects observed herein to HA preparations not induced by other undetected contaminations.

On the whole, all tested MW HA preparations stimulated the complex activation of blood phagocytes that is directly related to host defense against pathogens [8, 33], particularly pathogens expressing HA capsule. However, under pathological conditions of “sterile” inflammation in the absence of a pathogen, the ROS overwhelmingly produced by activated phagocytes could significantly contribute to the damage of the surrounding tissue. The degranulation accompanied by the increase in surface receptor expression is directly associated with the interaction of phagocytes with endothelium. Finally, the resulting proinflammatory potential of HA in blood, particularly LMW HA, is supported by a significant induction of TNF-*α*, one of the key cytokine in the development of inflammatory reactions. Thus, the activation of blood phagocytes by HA, particularly by LMW HA, could significantly contribute to pathological inflammatory processes.

## 5. Conclusions

Our findings provide new information about the complex activation of blood phagocytes by HA of different MW, which significantly contributes to our understanding of the regulation of inflammatory processes by HA. In contrast to other studies, we used highly purified HA of a pharmacological grade with a low polydispersity index. Data support the current view on LMW HA as a proinflammatory agent with a strong potency for activating blood phagocytes. However, since all the tested HA of different MW induced the complex activation of blood phagocytes, the data did not confirm the effects of HMW as an anti-inflammatory agent inhibiting blood phagocyte activation. However, more research is needed, employing highly purified HA of precisely defined MW, to fully elucidate the underlying mechanisms of the role of HA of different MW in inflammatory processes.

## Abbreviations

HA: Hyaluronan
HBSS: Hanks balanced salt solution
HMW: High molecular weight
LMW: Low molecular weight
OZP: Opsonized zymosan particles
PMA: Phorbol 12-myristate 13-acetate
PMNL: Polymorphonuclear leukocyte
ROS: Reactive oxygen species
TNF-*α*: Tumor necrosis factor-*α*.
